# Riding the waves: A study of retrun spillovers and inter-sector linkages in US equity markets during the COVID-19 pandemic

**DOI:** 10.1016/j.heliyon.2024.e25203

**Published:** 2024-01-29

**Authors:** Umar Kayani, Ahmet Faruk Aysan, Mrestyal Khan, Maaz Khan, Farrukh Nawaz

**Affiliations:** a College of Business, Al Ain University, Abu Dhabi, United Arab Emirates; b College of Islamic Studies, Qatar Foundation, Hamad Bin Khalifa University, Qatar; c Department of Management Sciences, Balochistan University of Information Technology, Engineering, & Management Sciences (BUITEMS), Quetta, Pakistan; d Department of Management Sciences, COMSATS University Islamabad, Islamabad, Pakistan; e Faculty of Business Studies, Arab Open University (AOU), Riyadh, Kingdom of Saudi Arabia

**Keywords:** COVID-19, U.S sector indices, Returns, Spillovers, DY methodology

## Abstract

In times of crisis, stock markets experience a significant increase in return volatility, which leads to spillovers across equity sectors. The purpose of this study is to investigate the asymmetric spillovers across ten U.S. equity sectors, representing different industries. Daily prices of sector indices were collected from 02 January 2018 to 22 October 2021 for the analysis. In addition, the study applied Diebold and Yilmaz's (2012) dynamic spillover methodology, along with the static and rolling windows phenomena, to examine the daily returns spillovers across sector indices. The results indicate that 82 % of volatility forecast error variance in U.S sector indices is due to the spillover effect. Moreover, both industrials and financials exhibit the highest gross spillovers to other sectors, while they also receive the highest spillovers from other sectors. Furthermore, the oil and gas sector and utilities sector receive the highest net returns spillovers. These empirical findings provide crucial information regarding the interdependence of U.S. sector indices during the COVID-19 pandemic, which is relevant for investors and practitioners.

## Highlights

• Study investigates asymmetric spillovers across ten U.S. equity sectors during the COVID-19 pandemic.
• Dynamic spillover methodology was applied to analyze daily returns spillovers across sector indices.
• Results show 82 % of volatility forecast error variance in U.S. sector indices is due to spillover effect.
• Industrials and financials exhibit the highest gross spillovers, while oil & gas and utilities sectors receive the highest net returns spillovers.

## 1 Introduction

The outbreak of COVID-19, originating from Wuhan, China, quickly turned into a global crisis. As of March 11, 2020, the virus had infected over 1 million people worldwide, resulting in thousands of deaths [1]. Likewise, the World Health Organization (WHO) declared COVID-19 a “global pandemic.” The spread of the virus has continued unabated, and as of January 29, 2022, the number of infected individuals has risen to 370, 878, 130, with 5,669,541 deaths caused by COVID-19. The pandemic has not only resulted in numerous deaths and infections but has also instilled fear and panic among people worldwide. As a consequence, it has created an emergency, and the spread of panic has been faster than the virus itself [2].

The COVID-19 pandemic has evolved into a catastrophic event, extending beyond the healthcare emergency to cause significant economic downturns [3,4]. Experts have deemed it the ‘Great Lockdown recession’ in economic history, given its global spread and impact, reminiscent of the 1930s ‘Great Depression.’ For instance, the United States' GDP contracted by 5 percent between Q4 2019 and Q4 2020 due to COVID-19. The pandemic has also increased the US unemployment rate from 4.4 % in March 2020 to 14.7 % and 13.3 % in April and May 2020, respectively. Additionally, investor panic triggered significant stock market crashes globally, including in the United States, where major indices like the DJ industrial average plummeted by 7.79 % on March 9, 2020, and 9.9 % on March 12, 2020, marking some of the most substantial drops in US history.

The interest in the spillover of risk among financial markets has increased due to recent financial crises [[5], [6], [7], [8], [9], [10], [11]]. The spillover effect refers to the transmission of cross-information across financial markets or assets, indicating their interconnectedness. Recognizing spillover as a significant determinant in calculating portfolio risk, it has become a crucial component in financial risk management [[12], [13], [14], [15], [16]]. Therefore, researchers have made meaningful efforts to comprehend and anticipate spillovers throughout the financial system, providing useful inputs for risk managers to construct diversified asset portfolios.

Furthermore, during times of financial crisis, particular focus is diverted to the risk spillovers, because financial markets tend to be more correlated during these financial stress periods. Moreover, the global financial crisis (GFC) 2008–2009, and the European debt crisis (EDC) 2010–2012 provide evidence in favor of this behavior [[17], [18], [19], [20]]. Currently, financial markets are confronted with extreme conditions in the form of a COVID-19 pandemic, hence it is essential to unfold the patterns of the return spillovers across U.S sector indices for the benefit of policymakers, and investors. Moreover, financial markets are the determinants of a dynamic financial system that is characterized by time-varying asymmetric dependence [21]. Thus, the correlation among financial assets is stronger in the bearish trend than in the bullish one. As a result, investors react more to negative shocks rather than the positive ones hence, leading to a decrease in portfolio diversification [22].

Transmission of the information in the form of volatility spillovers has been a point of attention of recent studies concerning U.S equities [21], commodities [[23], [24], [25]], cryptocurrencies [26,27], and currencies [28]. But still, financial literature lack empirical evidence regarding the returns spillover across the U.S equities sector especially, during the transmission of COVID-19. In addition to this, the U.S stock market being the largest across the globe is among the heavily influenced countries by the COVID-19 pandemic. Though, U.S equity market has gone through heavy financial pressures such as GFC, EDC etc. However, financial literature is limited with respect to investigating the footprints of the non-financial crisis, such as COVID-19 on the return spillovers across U.S sector indices.

Researchers in the past have examined the volatility transmissions across equity markets [[29], [30], [31], [32], [33], [34], [35], [36], [37]], FOREX markets [28,[38], [39], [40]], and among commodities [[41], [42], [43]]. However, there is very limited financial literature available in exploring the asymmetric return spillovers across U.S equity sectors during the COVID-19 crisis.

Furthermore, the layout of this paper is structured as follows. Section 2 consists of literature review, section 3 comprises of the dataset, and applied methodology. Empirical findings are given in section 4. At last, section 5 includes the conclusion.

## 2 Literature review

The literature regarding the risk spillovers has shown a rapid evolution, due to the onset of GFC (2008–2009) which ultimately resulted in contagion in global stock markets. Though the existing body of knowledge on volatility spillovers among global financial markets is extensive, but the studies concerning the sectors are very few. Hassan and Malik [44] in their study, have used the multivariate GARCH model in order to investigate the volatility spillovers across U.S equity indices. The empirical findings showed the presence of dynamic shocks, and risk transmission across the equity sectors.

Hammoudeh, Yuan [45] explored the risk spillovers among Gulf countries sector markets. The results showed risk spillovers on the sectoral level except the Qatar sector market. Moreover, Chen, Li [46] determined the non-symmetric volatility spillovers across the Chinese sectors. The findings revealed the presence of asymmetric risk spillovers among the sectors. Wu, Zhang [47] also analyzed the risk transmission among stock sector indices of the Chinese equity market via applying graph theory and VaR method. Their statistical findings showed that Chinese sector market is heavily dominated by industrial sector.

In the empirical literature, there has been a diverse range of models that are applied to model the volatility spillovers across the various financial markets at global level. Such as, Jebran, Chen [48] analyzed the volatility spillovers among Asian equity markets, during the GFC via using the E-GARCH approach. The empirical findings showed that during the crisis period the volatility spillover between Indian, and Sri Lankan markets is bi-directional. Whereas, in post-crisis times the equity markets of Pakistan and Sri Lanka showed bidirectional volatility spillover. Furthermore, Xu, Li [49] using the GARCH-X model examined the volatility spillovers between Shanghai equities, and Hong Kong equities. The authors figure out that mostly the volatility spillovers are bidirectional, and significantly increase during major financial happenings. Similarly, using the methodology of [50,51], Mensi, Boubaker [17] determined the risk spillovers among GIPSI equities. They found that during times of financial crisis, spillovers among stock markets gets increase. Based on the same methodology, Nishimura, Tsutsui [52] linked stock market volatility and returns transmissions with global stock investors' activities. The findings suggested that spillover of stock returns and volatility increase with rise in stock market openness.

During the periods of financial stress, special attention is given to spillovers, the reason being intensification in such times, GFC (2008–2009), and EDC (2010–2012) provide evidence in favor of this characteristic [17,18]. Furthermore, black swan events in a form of epidemics have caused panic among international investors [53]. For instance, Nippani* and Washer [54] in their study found unfavorable consequences of SARS epidemic on Vietnamese and Chinese equity markets. Moreover, on sectoral level Chen, Jang [55], found that during the SARS outbreak the stocks of the hotel industry in China and Taiwan gave negative returns. Similarly, Ebola another infectious disease in 2003, caused investors to resists buying equity in African [56], and U.S [57] equity markets.

Recently, researches have also explored the consequences of COVID-19 on several financial markets, globally. According to Baker, Bloom [58], COVID-19 as compared to previous pandemics had a significantly higher effect on the U.S equities. Similarly, the findings of Nicola, Alsafi [59], confirm the adverse results of COVID-19 on global financial markets. Moreover, as compared to Asian and Australian equity markets, the equity market of the USA has been more reactive during this global pandemic [60]. Further, Garcin, Klein [61] also reported similar results. Whereas, in comparison with the European stock markets, Asian stock markets are heavily influenced by COVID-19 crisis [62].

In recent times, the empirical literature has reported the drastic financial effects of COVID-19 based on various statistical models [58,[63], [64], [65], [66], [67], [68], [69], [70]]. Moreover, Yarovaya, Elsayed [71] in their study incorporated the DJ world index and Islamic stock index. The findings showed that during the COVID-19 crisis Islamic bonds (Sukuk) acts as safe haven instruments. Furthermore, Hung [72] investigated the volatility spillovers between prices of crude oil and five equity markets of Europe, and found increase in the volatility spillovers during COVID-19 outbreak.

Furthermore, recent evidence from Amar, Belaid [73] confirms the presence of increased spillovers across the equity markets during the COVID-19 crisis, along with the tendency of developed stock market to lead the developing stock markets. Likewise, studies show the risk spillovers effect from equity market of United States to four major African equity markets and across the global equity markets, respectively [74,75]. Meanwhile, the findings from the study of Fu, Liu [76] depicts that contagion effect was stronger for countries with stronger outbreaks. Moreover, Harjoto, Rossi [77] in their study found that the stock markets across the globe negatively reacted towards COVID-19 crisis.

Additionally, this study contributes to the existing literature on the risk spillovers in three ways. At first, the study explores the asymmetric return spillovers across ten different U.S sectors. Secondly, the study examines the spillovers caused by the COVID-19 across the selected equity sectors. Thirdly, the study applied Diebold and Yilmaz [50] spillover index method on the daily returns data set of sectors indices returns.

## 3 Data & methodology

### 3.1 Dataset

An increase in financial integration both on the sector and global level, has confronted financial markets and sectors with important challenges. Thus, it is evident to examine the directional return spillovers during the ongoing COVID-19 crisis on a sectoral basis, because it can give sufficient inputs to investment managers regarding which sectors are dominant with respect to affecting other sectors. For this purpose, data from all the sector markets of United States was used. Hence, this study attempts to investigate the return spillovers across the U.S equity sector indices during the COVID-19 crisis.

On March 11, 2020 W H.O declared COVID-19 as a “Global Pandemic.” Thus, based on the announcement date, this study divides the entire dataset ranging from 02-January-2018 to 22-October-2021 into two periods; 1) before the COVID-19 crisis (02-January-2018 to 10-March-2020); 2) during the COVID-19 crisis (11-March-2020 to 22-October-2021). The data sampled included the daily prices of ten U.S sector indices. A list of the selected U.S sector indices along with their respective ticker codes, lauch date, and characteristics are illustrated in Table 1.

**Table 1:** List of U.S sector indices along with their ticker codes, launch date, and characteristics.

S.No.	Sector Indices	Ticker codes	Launch Date	Index Characteristics
1	Dow Jones Basic Materials	(DJUSBM)	Feb 14, 2000	Number of constituents = 37 Total Market Capitalization (USD million) = 23,392.82
2	Dow Jones Consumer Goods	(DJUSNC)	Dec 20, 2004	Number of constituents = 106 Total Market Capitalization (USD million) = 34,965.25
3	Dow Jones Consumer Services	(DJUSCY)	Dec 20, 2004	Number of constituents = 141 Total Market Capitalization (USD million) = 38,988.81
4	Dow Jones Financials	(DJUSFN)	Feb 14, 2000	Number of constituents = 224 Total Market Capitalization (USD million) = 29,999.35
5	Dow Jones Health Care	(DJUSHC)	Feb 14, 2000	Number of constituents = 130 Total Market Capitalization (USD million) = 42,304.5
6	Dow Jones Industrials	(DJUSIN)	Feb 14, 2000	Number of constituents = 210 Total Market Capitalization (USD million) = 23,445.13
7	Dow Jones Oil & Gas	(DJUSEN)	Feb 14, 2000	Number of constituents = 41 Total Market Capitalization (USD million) = 46,244.63
8	Dow Jones Technology	(DJUSTC)	Feb 14, 2000	Number of constituents = 155 Total Market Capitalization (USD million) = 90,244.63
9	Dow Jones Telecommunications	(DJUSTL)	March 23, 2007	Number of constituents = 41 Total Market Capitalization (USD million) = 21,227.65
10	Dow Jones Utilities	(DJUSUT)	Feb 14, 2000	Number of constituents = 49 Total Market Capitalization (USD million) = 20,646.63

Furthermore, for empirical analysis, all the sector indices prices were converted into returns using the formula of continuously compounding given in equation.

(1) $$R_{t}=\ln\left(\frac{P_{t}}{P_{t-1}}\right)$$

where stock returns at current time period are represented by $R_{t}$, log natural function is denoted by ln whereas, $P_{t}$ and $P_{t-1}$ reflects the prices of stock at time period t, and t−1.

### 3.2 Methodology

We investigate the financial return spillovers across the equity sectors of the United States by adopting the Diebold and Yilmaz [50] spillover framework which based on the variance decomposition and associated with the n-variable vector autoregressive model. The DY spillover framework concentrate on the total spillover. This model provides significant advantage by estimating directional spillovers.

Consider a covariance stationary n-variable ${VAR}_{\left(p\right)}$ which is represented by:

(2) $$x_{t}=\sum_{i=1}^{p}\Phi_{i}x_{t-1}+\varepsilon_{t}$$

where ε∼(0,∑) is an independently and identically disturbances vector. The MA representation can be written as $x_{t}=\sum_{i=0}^{\infty }A_{i}\varepsilon_{t-i}$, where the n x n indicates the coefficient matrices of $A_{i}$, that follow the recursion $A_{i}=\Phi_{1}A_{i-1}+\Phi_{2}A_{i-2}+\ldots +\Phi_{p}A_{i-p}$, with $A_{0}$ is an identity matrix and $A_{i}=0$ for *I* < 0. The dynamic of the system can be recognized by coefficients of the moving average. The entire procedure is dependent upon the variance decompositions that assist us to examine the forecast error variance of each repeated variable into the segment that is highly associated with shocks in the system. Therefore, with assistance of variance decomposition, we are able to estimate the H-step-ahead error variance in the process of detection of $x_{i}$ because of shocks to $x_{j},\forall j\neq i$ for each i.

The KPPS H-step-ahead is denoted by $\theta_{ij}^{g}\left(H\right)$, where H=1,2,…, so we have

(3) $$\theta_{ij}^{g}\left(H\right)=\frac{\sigma_{jj}^{-1}\sum_{h=0}^{H-1}{\left(e_{i}^{\prime }A_{h}\sum e_{j}\right)}^{2}}{\sum_{h=0}^{H-1}\left(e_{i}^{\prime }A_{h}\sum e_{j}\right)}$$

whereas $\sigma_{jj}$ denotes the standard deviation of the error term for the *j*th equation. ∑ shows the variance matrix of error term. Furthermore, The selection vector is denoted by the $e_{i}$, equal to one for the ith element and zero otherwise.

In each row, the sum of elements of variance decomposition is not equal to 1, so it can be written as $\sum_{j=1}^{N}\theta_{ij}^{∼g}\left(H\right)$. Therefore,

(4) $$\theta_{ij}^{∼g}\left(H\right)=\frac{\theta_{ij}^{g}\left(H\right)}{\sum_{j=1}^{N}\theta_{ij}^{g}\left(H\right)}$$

which is applied in the spillover index computation, $\sum_{j=1}^{N}\theta_{ij}^{∼g}\left(H\right)=1$ and $\sum_{i.j=1}^{N}\theta_{ij}^{∼g}\left(H\right)=N$. The contributions of volatility in the development of the total spillover index is derived by KPPS variance decomposition:

(5) $$S^{g}\left(H\right)=\frac{\sum_{\begin{array}{c} i,j=1\\ i\neq j \end{array}}^{N}\theta_{ij}^{∼g}\left(H\right)}{\sum_{ij=1}^{N}\theta_{ij}^{∼g}\left(H\right)}∙100=\frac{\sum_{\begin{array}{c} i,j=1\\ i=j \end{array}}^{N}\theta_{ij}^{∼g}\left(H\right)}{N}∙100$$

The overall spillover shocks have been represented by total spillover index. Furthermore, we computed the directional spillover properties to the market *i* for all other markets *j* as:

(6) $$S_{i.}^{g}\left(H\right)=\frac{\sum_{\begin{array}{c} j=1\\ j\neq i \end{array}}^{N}\theta_{ij}^{∼g}\left(H\right)}{\sum_{i,j=1}^{N}\theta_{ij}^{∼g}\left(H\right)}∙100=\frac{\sum_{\begin{array}{c} j=1\\ j\neq i \end{array}}^{N}\theta_{ij}^{∼g}\left(H\right)}{N}∙100$$

Similarly, we also computed the directional spillover by the market *i* to all other *j* markets:

(7) $$S_{.i}^{g}\left(H\right)=\frac{\sum_{\begin{array}{c} j=1\\ j\neq i \end{array}}^{N}\theta_{ji}^{∼}\left(H\right)}{\sum_{i,j=1}^{N}\theta_{ji}^{∼g}\left(H\right)}∙100=\frac{\sum_{\begin{array}{c} j=1\\ j\neq i \end{array}}^{N}\theta_{ji}^{∼g}\left(H\right)}{N}∙100$$

The net spillover for the sectors has been estimated by the above equation which provides the overall summary of information of each variable.

(8) $$S_{i}^{g}=S_{.i}^{g}\left(H\right)-S_{i.}^{g}\left(H\right)$$

Furthermore, the net pairwise spillover of the sector indices returns can be written as:

(9) $$S_{ij}^{g}\left(H\right)=\left(\frac{\theta_{ji}^{∼g}\left(H\right)}{\sum_{i,k=1}^{N}\theta_{ik}^{∼g}\left(H\right)}-\frac{\theta_{ij}^{∼g}\left(H\right)}{\sum_{j,k=1}^{N}\theta_{jk}^{∼g}\left(H\right)}\right)∙100=\left(\frac{\theta_{ji}^{∼g}\left(H\right)-\theta_{ij}^{∼g}\left(H\right)}{N}\right)∙100$$

### 3.3 Descriptive statistics

Table 2, illustrates the descriptive statistics for ten U.S equity sectors. All the sectors indices exhibit positive mean returns except Oil & Gas, and Telecommunications. Moreover, Oil & Gas gives the highest value for the standard deviation whereas, Consumer Goods gives the lowest standard deviation value however, the values for the selected sectors indices range from 0.01 to 0.02. Furthermore, the values of skewness for sectors returns are negative which means that the distribution of returns series for all sectors is skewed negatively. Whereas, the statistics for kurtosis show that sector indices return follow the leptokurtic phenomena. Additionally, the results extracted from normality test (Jarque-Bera) showed that the returns series of all the sector indices are asymmetrically distributed. Moreover, Table 2 also represents the Augmented Dickey-Fuller (ADF) test results; it can be noted that the ADF values for each of the sector indices returns are significant at 1 % level. Hence, the returns series of the selected sector markets is stationary, rejecting the hypothesis of the presence of unit root.

**Table 2:** Descriptive statistics of U.S Sectors Indices.

Sector Indices	Mean	Median	Std. Dev	Kurtosis	Skewness	Min	Max	Jarque-Bera Test	ADF Value
DJUSUT	0.0002	0.0008	0.0146	19.4739	−0.2599	−0.1224	0.1222	2877.6***	−15.069***
DJUSTC	0.0009	0.0020	0.0171	10.3868	−0.7584	−0.1460	0.1069	2309.8***	−16.337***
DJUSEN	−0.0002	0.0001	0.0236	16.2680	−1.0788	−0.2317	0.1487	520.57***	−14.75***
DJUSIN	0.0004	0.0012	0.0153	15.0127	−0.8985	−0.1332	0.1139	2973.1***	−14.51***
DJUSHC	0.0005	0.0008	0.0127	11.4852	−0.5737	−0.1082	0.0733	2770.1***	−14.899***
DJUSFN	0.0004	0.0010	0.0163	20.0033	−1.1184	−0.1585	0.1133	3403.5***	−14.587***
DJUSCY	0.0006	0.0014	0.0133	13.6066	−1.2028	−0.1171	0.0725	3617***	−15.435***
DJUSNC	0.0004	0.0010	0.0125	14.9070	−1.1222	−0.1054	0.0767	2607.4***	−15.218***
DJUSBM	0.0002	0.0006	0.0164	10.2031	−0.6908	−0.1147	0.1135	1116.4***	−14.414***
DJUSTL	−0.0001	0.0007	0.0126	7.8570	−0.3454	−0.0856	0.0789	1797***	−15.217***

Note: *** represents 1 % significance level, ** refers to 5 % significance level, * shows 10 % significance level.

Table 3 refers to the pearson correlation matrix for all the 10 U S equity sector markets during the COVID-19 crisis. Overall, it can be noted that the whole set of pairs of correlations (100) are positive during the COVID-19 pandemic. Moreover, we observed the strongest positive association between the pairs of DJUSFN and DJUSIN, DJUSBM and DJUSIN with a correlation values of 0.95 and 0.93, respectively followed by the pairs of DJUSCY and DJUSTC (0.91), DJUSBM and DJUSFN (0.90). Furthermore, it can be seen that the weakest positive association exists between the pairs of DJUSEN and DJUSTC (0.48), DJUSEN and DJUSUT (0.51) followed by the pairs of DJUSHC and DJUSEN (0.56), DJUSTL and DJUSEN (0.58).

**Table 3:** Pearson Correlation matrix for all the ten equity sectors during the COVID-19 pandemic.

Equity Sectors	DJUSUT	DJUSTC	DJUSEN	DJUSIN	DJUSHC	DJUSFN	DJUSCY	DJUSNC	DJUSBM	DJUSTL
DJUSUT	1.00	0.62	0.51	0.78	0.77	0.78	0.68	0.79	0.71	0.77
DJUSTC	0.62	1.00	0.48	0.77	0.81	0.69	0.91	0.83	0.68	0.59
DJUSEN	0.51	0.48	1.00	0.77	0.56	0.81	0.60	0.61	0.79	0.58
DJUSIN	0.78	0.77	0.77	1.00	0.83	0.95	0.85	0.88	0.93	0.76
DJUSHC	0.77	0.81	0.56	0.83	1.00	0.78	0.79	0.83	0.75	0.73
DJUSFN	0.78	0.69	0.81	0.95	0.78	1.00	0.80	0.83	0.90	0.78
DJUSCY	0.68	0.91	0.60	0.85	0.79	0.80	1.00	0.86	0.77	0.68
DJUSNC	0.79	0.83	0.61	0.88	0.83	0.83	0.86	1.00	0.81	0.75
DJUSBM	0.71	0.68	0.79	0.93	0.75	0.90	0.77	0.81	1.00	0.71
DJUSTL	0.77	0.59	0.58	0.76	0.73	0.78	0.68	0.75	0.71	1.00

## 4 Empirical analysis

**Fig. 1:**
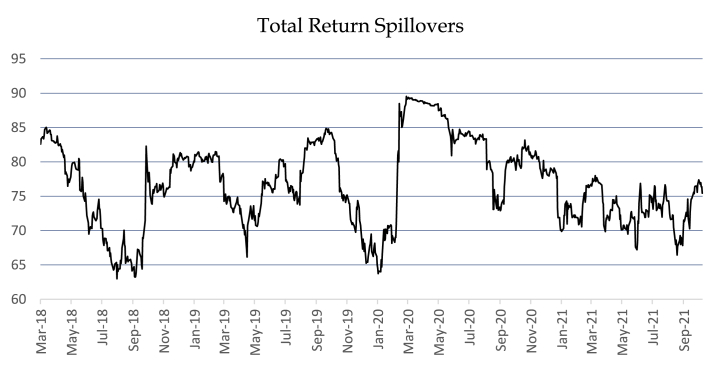
Total Return Spillovers for all the ten Sector Indices in times of COVID-19.

Fig. 1 depicts the total return spillovers for all the ten U.S sector indices in times of COVID-19 crisis. The entire data is split into two phases: the phase before the COVID-19 crisis (02-January-2018 to 10-March-2020), and the phase during the COVID-19 crisis (11-March-2020 to 22-October-2021). Moreover, it can be noted that before the declaration of the pandemic, there is a significant decrease in the spillovers among sector indices from a maximum value (88.48) to a minimum value (62.98). Whereas, during the COVID-19 phase there has been a slight increase in the spillovers across the ten sector indices with a minimum value (66.44), and maximum value (89.52).

### 4.1 Rolling window analysis in regimes

To understand the mechanism of spillovers across U.S equity markets, we analyze the spillover index during two regimes (Pre-COVID-19 crisis, and During COVID-19 crisis). Fig. 1 illustrates the dynamic nature of the total spillover index based on 100-day rolling window. Overall, two regime periods were identified. As shown, in the pre-COVID-19 crisis the total spillover index initiated with approximately 85 percent, and increased to almost 90 % at the end of February 2020, due to sharp rise in COVID-19 infectants. Whereas, during COVID-19 crisis, the index begins with around 90 percent, and remains between 80 percent to 90 percent from March 2020 to August 2020, probably because of the immense rise in the COVID-19 cases across the world, particularity in the United States of America. However, the total spillover index declines up to 75 % later at the end of the period due to lucrative economic policies adopted by the U.S and the start of the vaccination against COVID-19 in the U.S.

However, it can be seen that there is not much difference in the spillovers between the two periods (before the pandemic, and during the pandemic). Therefore, we applied *t*-test for the means as shown in Table 4, which shows the variance for sector indices before the COVID-19 increased from (35.78) to (36.24) during the COVID-19. Hence, it can be seen that there is an increase in spillovers during the pandemic phase as compared to before the pandemic phase. However, in order to nullify the increase in the spillovers during crises times, the policymakers must ensure the ease of access of liquidity to the financial markets [78]. Additionally, all the pairwise spillovers graphs for each of the sectors pairs are given in Appendix A section in detail.

**Table 4:** t-test: Assuming Unequal Variances.

Particulars	Before	During
Mean	75.59013	78.02046
Variance	35.78332	36.24806
Minimum	62.98596	66.44721
Maximum	88.48487	89.52756
Observations	500	410
Hypothesized Mean Difference	0	
df	871	
t Stat	−6.07628	
P(T ≤ t) one-tail	9.18472E-10	
t Critical one-tail	1.64660	
P(T ≤ t) two-tail	1.83694E-09	
t Critical two-tail	1.96269	

### 4.2 Dynamic spillovers across U.S equity sectors

**Fig. 2:**
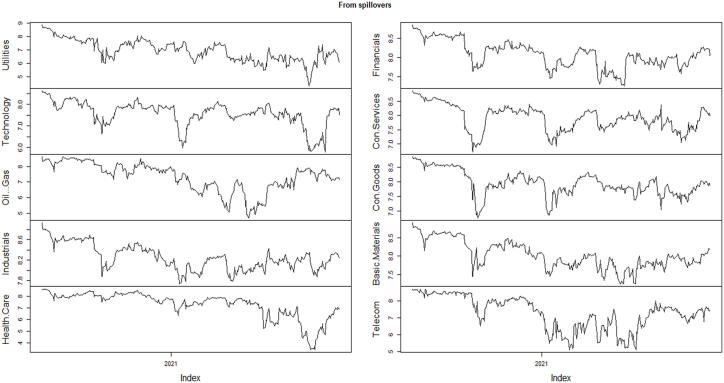
Directional spillovers, from all sector indices returns during COVID-19.

Fig. 2 and Table 5 represent the directional spillovers from each of the ten sector indices to the other sectors, which is shown as “Directional TO others”. It can be seen that within the ten sector indices the gross spillovers from industrials (102.53), financials (98.92), and consumer goods (94.29) are greater than other sectors. Whereas, gross spillovers from oil & gas (53.88), utilities (68.75), and technology (74.09) are the lowest than other sectors.

**Table 5:** Total return spillovers matrix across U.S equity sectors.

Sector Indices	Utilities	Technology	Oil & Gas	Industrials	Health Care	Financials	Con Services	Con Goods	Basic Materials	Telecom	From
Utilities	18.47	6.82	4.04	10.46	10.85	10.82	8	10.86	8.45	11.24	81.54
Technology	5.85	18.4	3.92	10.24	11.82	8.43	14.67	12.18	7.7	6.78	81.59
Oil & Gas	5.04	4.64	22.03	12.39	6.82	13.99	7.04	7.79	12.82	7.44	77.97
Industrials	7.78	8.16	7.69	13.76	9.55	12.58	9.85	10.63	11.57	8.45	86.26
Health Care	8.83	10.92	4.47	10.65	17.13	9.52	10.15	11.06	8.48	8.78	82.86
Financials	8.3	6.76	8.96	12.9	8.87	14.81	9.05	9.87	11.38	9.1	85.19
Con Services	6.61	13.05	5.08	11.13	9.83	10.02	15.95	11.78	8.79	7.75	84.04
Con Goods	8.64	10.49	4.98	11.32	10.35	10.1	11.17	15.22	9.37	8.37	84.79
Basic Materials	7.14	6.95	9.02	13.12	8.69	12.55	8.79	10.1	15.49	8.16	84.52
Telecom	10.56	6.3	5.72	10.32	9.83	10.91	8.49	10.02	8.81	19.04	80.96
Directional TO Other	68.75	74.09	53.88	102.53	86.61	98.92	87.21	94.29	87.37	76.07	829.72
Directional Including Own	87.22	92.49	75.91	116.29	103.74	113.73	103.16	109.51	102.86	95.11	82.97 %
Net Directional Connectedness	−12.79	−7.5	−24.09	16.27	3.75	13.73	3.17	9.5	2.85	−4.89	82.97 %

**Fig. 3:**
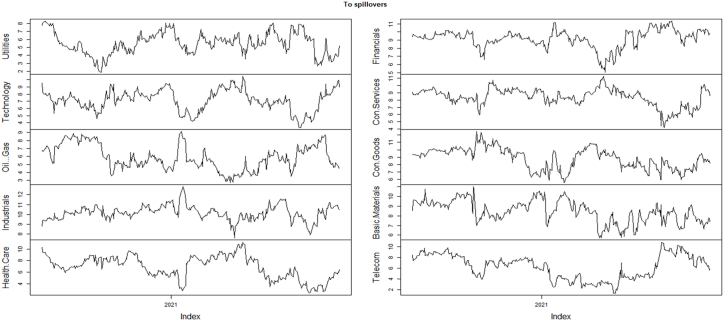
Directional spillovers, to all sector indices sectors during COVID-19.

Moreover, Fig. 3 illustrates the directional spillovers from the other sectors to each of the U.S sectors labeled as “Directional from other” in Table 5. During the pandemic, industrials followed by financials, and consumer goods with having values 86.26, 85.19, and 84.79 respectively, show the highest volatility spillovers. In contrast, oil & gas (77.97) sector followed by telecommunications (80.96), and utilities (81.54) show the lowest directional spillovers.

**Fig. 4:**
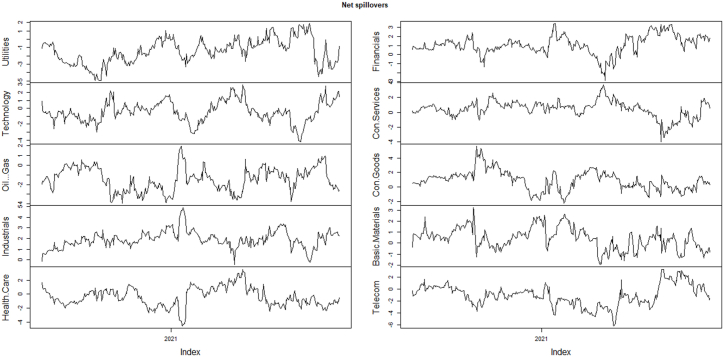
Net spillovers in sector indices returns during COVID-19.

Additionally, the net spillovers plot and values of U.S equity sectors are shown in Fig. 4 and Table 5. Every single point in Fig. 4 represents the net spillover of each equity sector which is equal to the difference between sums of the “Contribution from” column and “Contribution to” row. According to Fig. 4, during the COVID-19 crisis the net spillovers for most of the sectors remained negative particularly sectors including utilities, technology, oil & gas, and telecommunications however, net spillovers remained positive for sectors that include industrials, financials, and consumer goods. Moreover, in a nutshell out of ten sectors, six are net recipients, and four are net transmitters.

Overall, the statistical results presented in Table 4 shows that the total spillover is 82.97 %, which proves high level of connectedness across the ten U.S equity sectors. Hence, indicating the existence of spillover effect. Moreover, these results are in line with the findings of [44], confirming the presence of high level of spillover across U.S equity sector indices. Furthermore, in the context of directional spillovers transmitted ‘To’, Industrials (102.52) sector is the largest spillover transmitter to others whereas, Oil & Gas (53.88) sector is the lowest spillover transmitter to others. Both of these sectors also remained largest and smallest return spillovers recipients from others. Additionally, keeping in view net spillovers, Industrials sector with a value of 16.27 is regarded as the largest net transmitter of returns across the ten U.S sectors, followed by financials sector, and consumer goods sector. In contrast, oil & gas sector is identified as the highest net recipient of the spillovers among the ten sectors followed by utilities sector, and technology sector.

## 5 Conclusion

Keeping in view financial crises, especially COVID-19 it is essential to understand the transmission of returns spillovers across equity sectors during the times of the current pandemic. This paper made use of the methods of Diebold and Yilmaz (2012), to determine the net, and gross spillovers across the ten U.S equity sector indices using daily prices ranging from 02-January-2018 to 22-October-2021.

Furthermore, empirical findings are summarized as follows. Firstly, the results revealed significant connectedness across the U.S sector indices during the COVID-19 crisis thus, proving the existence of ‘spillover effect’. Secondly, we found that during the COVID-19 crisis, in U.S sector market Industrials sector followed by financials sector are identified as the major sources of spillovers that transmit risk to other equity sectors. Thirdly, findings also showed that during the COVID-19, both industrials sector, and financials sector were the largest recipients to the return spillovers in the U.S sector market. Fourthly, we observe that the total spillover index was high in both the periods (Before COVID-19, and During COVID-19 crisis) however, the volatility increased during the COVID-19 crisis. Finally, the study found that among all the selected sectors, utilities, technology, oil & gas, and telecommunications are net recipients of the returns transmitted by other sectors, whereas industrials, financials, health care, consumer goods, consumer services, and basic materials are the net transmitters.

In sum, the results reveal a significant interconnectedness among the U.S sector indices, with industrials and financials sectors being the major sources and recipients of spillovers, transmitting risk to other sectors. Moreover, the study found that utilities, technology, oil & gas, and telecommunications sectors are net recipients of the returns transmitted by other sectors, while industrials, financials, health care, consumer goods, consumer services, and basic materials are net transmitters. The findings of this study provide useful implications for policy-makers and investors regarding the transmission of financial shocks across equity sectors during the COVID-19 crisis. The findings reveal that U.S equity sectors exhibit a high level of interconnection and integration, leading to an increase in spillovers during the pandemic. Any changes in the prices of one equity sector's index can potentially result in fluctuations in the prices of other equity indices. The existence of spillovers among the ten U.S sector indices suggests that the possibility of low diversification during pandemic times, making it crucial for investors to be rational and cautious in their investments. Consequently, investors should consider investing in sectors that exhibit low return spillovers while keeping in view their risk appetite.

This study sets stage for future investigation. First, this study comprises of the sector markets of the United States, studies can include the equity sectors of highly infected countries by COVID-19 such as China, India, and Italy etc. Second, our study is limited to spillovers during the COVID-19, future researchers can also compare the results with the window of After COVID-19 pandemic. Third, the inclusion of explanatory factors such as interest rates and industrial production may assist in identifying the sources of asymmetry in volatility spillovers.

## Fundig statement section

Fundig statement Open Access funding provided by the Qatar National Library. This project/publication was supported by the CIS Research Clusters’ Grant.

## Data avalaiblitiy

Data used for the analysis in this study is availably on www.investing.com.

## CRediT authorship contribution statement

**Umar Kayani:** Writing – review & editing, Writing – original draft, Visualization, Validation, Supervision, Software, Resources, Project administration, Methodology, Investigation, Funding acquisition, Formal analysis, Data curation, Conceptualization. **Ahmet Faruk Aysan:** Writing – review & editing, Writing – original draft, Visualization, Validation, Supervision, Software, Resources, Project administration, Methodology, Investigation, Funding acquisition, Formal analysis, Data curation, Conceptualization. **Mrestyal Khan:** Writing – review & editing, Writing – original draft, Visualization, Validation, Supervision, Software, Resources, Project administration, Methodology, Investigation, Funding acquisition, Formal analysis, Data curation, Conceptualization. **Maaz Khan:** Writing – review & editing, Writing – original draft, Visualization, Validation, Supervision, Software, Resources, Project administration, Methodology, Investigation, Funding acquisition, Formal analysis, Data curation, Conceptualization. **Farrukh Nawaz:** Writing – review & editing, Writing – original draft, Visualization, Validation, Supervision, Software, Resources, Project administration, Methodology, Investigation, Funding acquisition, Formal analysis, Data curation, Conceptualization.

## Declaration of competing interest

The authors declare that they have no known competing financial interests or personal relationships that could have appeared to influence the work reported in this paper.

## Figures and Tables

**Fig. A1:**
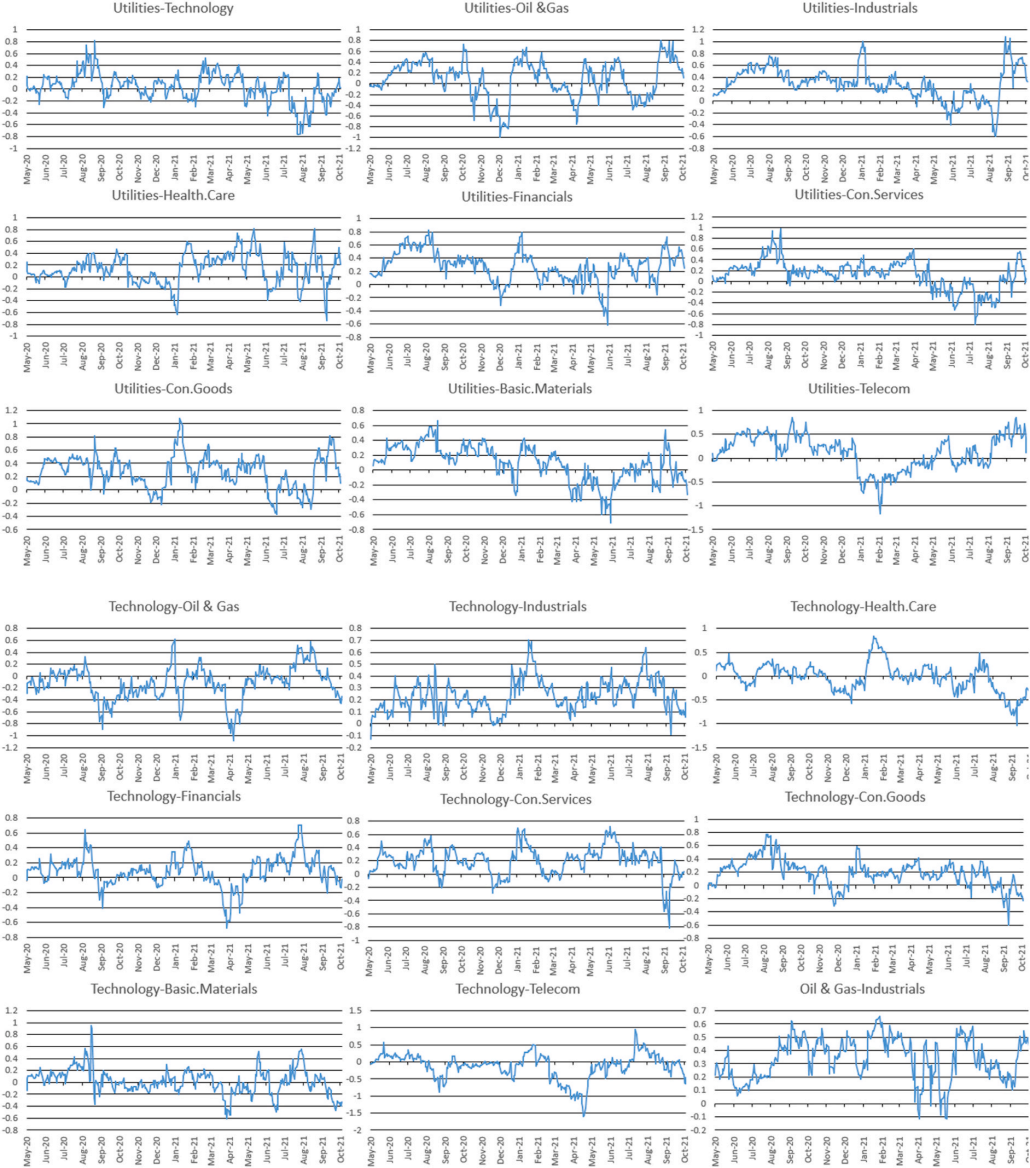
Pairwise Spillovers

**Fig. A2:**
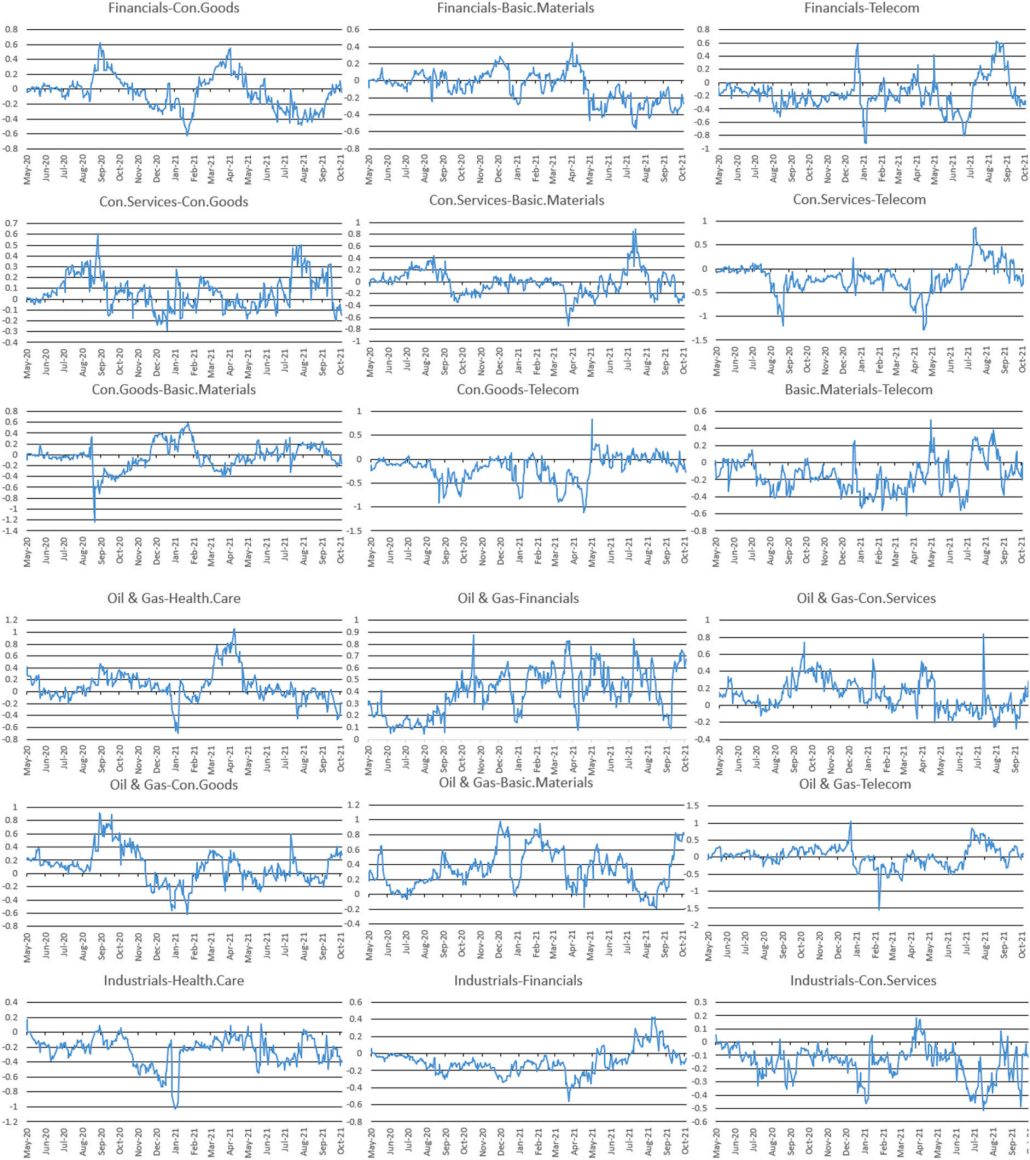
Pairwise Spillovers.

**Fig. A3:**
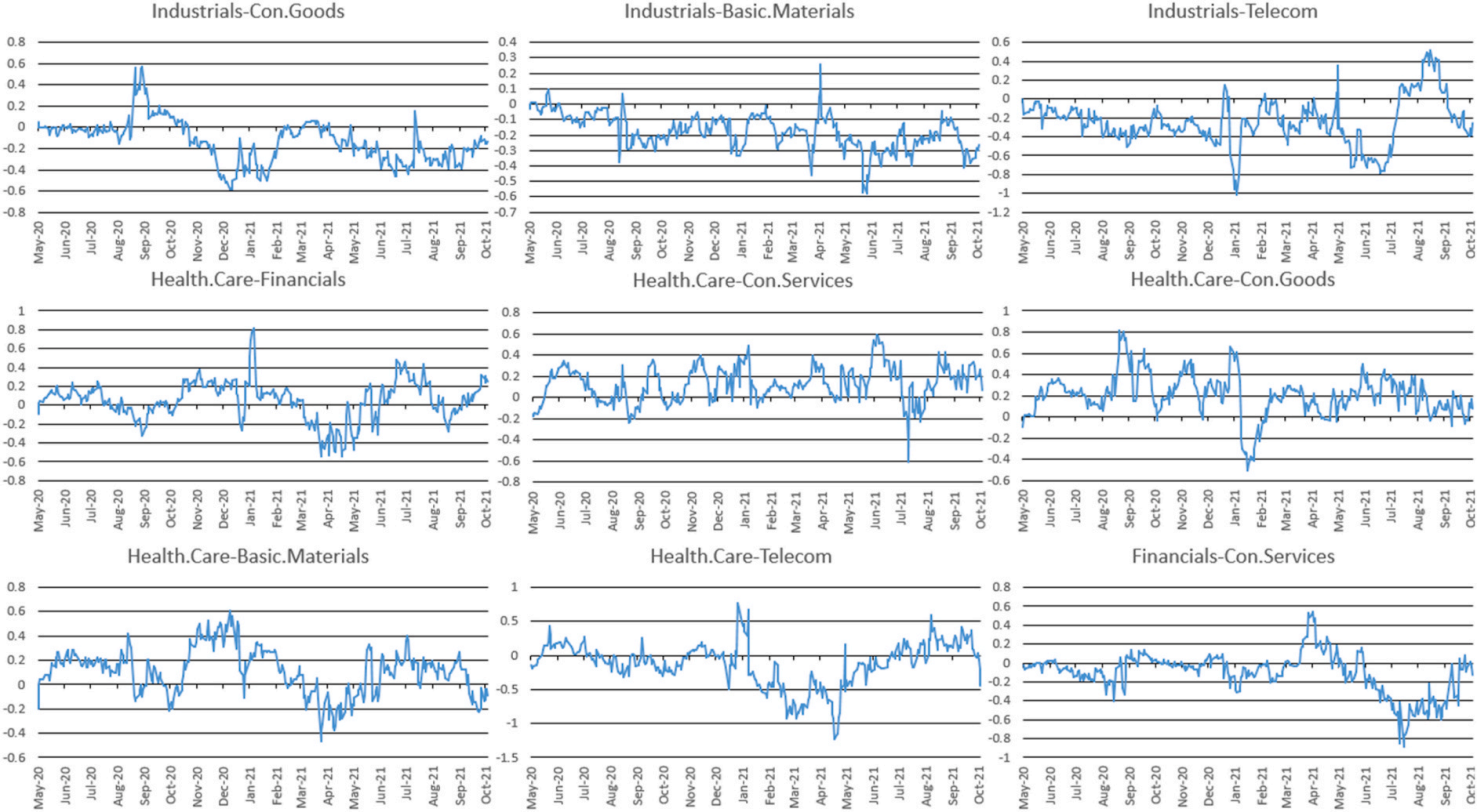
Pairwise Spillovers.
